# What is involved in medicines management across care boundaries? A qualitative study of healthcare practitioners' experiences in the case of acute kidney injury

**DOI:** 10.1136/bmjopen-2016-011765

**Published:** 2017-01-18

**Authors:** Denham L Phipps, Rebecca L Morris, Tom Blakeman, Darren M Ashcroft

**Affiliations:** 1 NIHR Greater Manchester Patient Safety Translational Research Centre, The University of Manchester, Manchester, UK; 2 Centre for Pharmacoepidemiology and Drug Safety Research, Manchester Pharmacy School, The University of Manchester, Manchester, UK; 3 Centre for Primary Care, Institute of Population Health, The University of Manchester, Manchester, UK; 4 NIHR Greater Manchester Collaborative for Leadership in Applied Health Reserach and Care, The University of Manchester, Manchester, UK

## Abstract

**Objectives:**

To examine the role of individual and collective cognitive work in managing medicines for acute kidney injury (AKI), this being an example of a clinical scenario that crosses the boundaries of care organisations and specialties.

**Design:**

Qualitative design, informed by a realist perspective and using semistructured interviews as the data source. The data were analysed using template analysis.

**Setting:**

Primary, secondary and intermediate care in England.

**Participants:**

12 General practitioners, 10 community pharmacists, 7 hospital doctors and 7 hospital pharmacists, all with experience of involvement in preventing or treating AKI.

**Results:**

We identified three main themes concerning participants' experiences of managing medicines in AKI. In the first theme, *challenges arising from the clinical context*, AKI is identified as a technically complex condition to identify and treat, often requiring judgements to be made about renal functioning against the context of the patient's general well-being. In the second theme, *challenges arising from the organisational context*, the crossing of professional and organisational boundaries is seen to introduce problems for the coordination of clinical activities, for example by disrupting information flows. In the third theme, *meeting the challenges*, participants identify ways in which they overcome the challenges they face in order to ensure effective medicines management, for example by adapting their work practices and tools.

**Conclusions:**

These themes indicate the critical role of cognitive work on the part of healthcare practitioners, as individuals and as teams, in ensuring effective medicines management during AKI. Our findings suggest that the capabilities underlying this work, for example decision-making, communication and team coordination, should be the focus of training and work design interventions to improve medicines management for AKI or for other conditions.

**Strengths and limitations of this study**

- The composition of the sample meant that participants represented practice across three different geographical areas of England.
- Semistructured interviews allowed the researchers to explore participants' experience of medicines management during acute kidney injury in depth.
- The data examine the regular work of practitioners independently of patient outcome, as opposed to examining behaviour during adverse events only.
- The findings may have limited transferability to other locations if the tasks discussed are organised or distributed differently elsewhere.
- No ‘first-hand’ observational data were available to compare with interview accounts.

## Background

Ensuring safe and effective medication usage in the context of acute illness can be a challenging activity; even more so when patient care occurs across different sectors, organisations or even departments or specialties. That difficulties in medicines management can be encountered under such circumstances is suggested by case note and incident reviews in Western Europe, the USA, Australia and Canada, which have found deficiencies in the transfer of medicines information and coordination of care activities during either admission to or discharge from the hospital.^1–7^ In addition, case note studies in the USA^8^ and Germany^9^ found hospitalisation to be a risk factor in itself for prescribing errors, with safety further compromised by poor communication between primary and secondary care about medication changes that occur while in hospital.

A number of studies have sought to examine the problems that occur during transition, and how these can be addressed. For example, process maps of information handling after transfer between primary and secondary care have identified opportunities for information to be incorrectly or incompletely recorded, or for it to not be shared with those care professionals who are responsible for acting on it.^10^ ^11^ These problems are believed to be the result of various gaps in medicines management, ranging from differences in the interpretation of institutional policy, through limitations in professional interworking, to difficulties in the execution and coordination of tasks at the front line by healthcare professionals and patients.^11^ ^12^ Hence, there are a number of approaches that can be taken to understanding and improving medicines management across care interfaces. Technical solutions include a system for organising, updating and sharing medicines information between care providers and patients.^12–14^ However, as a study of technology-mediated communication between pharmacists and physicians^15^ illustrates, the working relationship between healthcare professionals provides an important context for the collaborative use of medicines management tools. Alstveit *et al*^16^ suggest that at the interface between primary and secondary care, interaction can be hampered by poor communication, a focus on one's own tasks rather than the work system as a whole and a lack of clarity about the division of task responsibility. Meanwhile, Waring *et al*^17^ note that occupational and organisational boundaries between the parties involved in transitional care can exacerbate differences in knowledge and practice, hence adding to the complexity of medicines management.

Given these insights, medicines management in acute illness can be seen as an instance of cognitive work. In other words, it draws on the set of skills that enable practitioners to understand a complex and dynamic situation, decide how to act and then carry out the chosen actions.^18^ Where the activity is distributed between different actors or teams, as is the case in care transitions, an important aspect of cognitive work is sharing information and understanding of the information, in order to facilitate coordinated activity.^19^ An illustrative Australian study by Tariq *et al*^20^ examined medicines handling in residential aged care, and found that a major contributor to medication errors was the presence of gaps in information sharing. This was, in turn, attributed to aspects of work design that were not conducive to reliable communication and coordination across the specialties involved (eg, the design of medicines charts and the scheduling of activities). The study authors further noted that the care professionals involved attempted to address the information gaps by developing local practices on their own initiative, such as additional medicine checks or follow-up telephone calls.

To provide an appropriate system of work for medicines management across care boundaries,^21^ it is therefore useful to understand the experiences of those who carry out this activity in practice. The current paper focuses on medicines management during actual or suspected acute kidney injury (AKI); this being an exemplar of a complex clinical situation that crosses care boundaries. AKI is a clinical syndrome in which renal functioning rapidly deteriorates, potentially with a negative impact on the patient's general condition. While it can be a consequence of damage to the kidney itself, AKI often follows other conditions that impair either supply to, or output from, the kidney. It has been estimated to affect some 15% of all patients admitted to the hospital, with a mortality rate of ∼30%.^22^ ^23^ A review of AKI-related patient deaths in the UK^24^ noted that 14% of the cases were judged to be preventable in either primary or secondary care. Hence, AKI has implications for activities in a range of care settings.^25–27^

Among the recommendations made by the National Institute for Health and Clinical Excellence^22^ are several that concern the appropriate use of medicines when there is a risk of AKI, whether or not it has actually occurred. The medicines optimisation issues to consider include the suspension, continuation or adjustment of particular medicines, the monitoring of medication effects on renal function and the sharing of medicines information between primary and secondary care practitioners, as well as patients.^28^ While such issues may be straightforward in principle, evidence suggests that they are challenging to enact in practice; particularly so, when they involve interactions between primary and secondary care.

These problems can be understood in the context of the work that is required to manage medicine usage when there is a risk of AKI, and the circumstances under which such work occurs. Leaving aside preventative measures such as the interrogation of electronic health records,^29^ ^30^ the successful management of patients when AKI may be present depends on practitioners detecting the onset of AKI in the first place, and, second, intervening appropriately. In general terms, this could be described as an iterative process of observation (gathering data and detecting patterns of genuine importance), decision-making (determining whether there is a situation arising that requires a response and formulating a course of action), action (implementing the course of action) and re-evaluation (determining whether the action dealt with the situation and whether any side-effects have arisen).^31^

Hence, gathering and interpretation of data about the patient play an important role. In practice though, such work is often shared between primary and secondary care, and between practitioners within each setting. Such a situation also creates the need for coordination between the different parties involved. Espinosa *et al*^32^ and Rico *et al*^33^ suggest several means by which members of a team can coordinate their task activity. In broad terms, these are *explicit* (the use of plans, procedures or communications specifically intended to achieve coordination) and *implicit* (the general sharing of knowledge about the task or each other's activities) coordination. Either, or both, may be required to achieve collaborative work, depending on the circumstances of the task. In the context of renal care, the role of information handling and collaborative working in medicines management has been alluded to by studies of pharmacists^34^ and nephrologists.^35^ This suggests that transitional care related to renal conditions would benefit from a more in-depth understanding of the facilitators and barriers to practitioners' work.

The aim of the study reported in this study was to examine how the work of medicines management during actual or suspected AKI is achieved in practice. To address this question, we drew from the experiences of healthcare practitioners in the UK who are involved in managing medicines for patients at risk of AKI, in particular when their work crosses care boundaries.

## Method

### Study design and setting

Our study used a qualitative design. It was informed by a realist epistemology, in which a participant's experiential account is seen to provide an interpretation of the social and technical processes that create his or her work setting.^36^ The perspective from which we elicit and read participants' accounts in this study is that of health services researchers with an interest in quality and safety improvement and, for two of the four researchers, a professional background that relates to the study topic (pharmacy or general practice). All of the researchers had previous experience of conducting qualitative health services research at doctoral level.

The sampling frame was primary and secondary healthcare practitioners involved in managing medicines for patients at risk of AKI. Data collection took place in three geographical areas of England. In the North West of England, the study sites were three teaching hospitals with 800 beds, 950 beds and 700 beds, respectively, a primary care administrative region serving 216 000 patients and an intermediate care facility of 30 beds. The latter facility was for patients who have been discharged from hospital but need further care before returning to the community. In the East Midlands, the study sites were one teaching hospital with 1100 beds and one primary care administrative region serving 546 000 patients. In the South-West of England, the study site was a primary care administrative region serving 550 000 patients. All of the study sites provide general healthcare services and therefore encounter patients with various healthcare conditions, including AKI. At the time of data collection, the primary care sites were involved in a separate study by two of the study authors (RLM and TB) to evaluate an AKI prevention tool;^37^ the qualitative data from this evaluation form part of the data set for the current study.

### Participants

Participants were initially recruited to the study using purposive sampling; we sought to include elements of the sampling frame that represented the different roles responsible for medicines management in AKI (doctors and pharmacists) and the different settings within which AKI management takes place (primary, secondary and intermediate care). We identified relevant members of the sampling frame through local research and professional networks and personal contacts, and invited them by email to take part in an interview about the management of AKI. To obtain a broader range of views, we then used snowball sampling to extend the study sample, with healthcare professionals already recruited nominating colleagues at their respective sites for us to approach. One person who we approached was unavailable for interview, and a second agreed to take part but then had to withdraw due to illness. Participant recruitment continued until no new insights were obtained from interviews. The composition of the final sample is shown in table 1.

**Table 1:** Participant roles and locations

Location	Participant's role N
Primary care	
North-West England	General practitioner 7
	Community pharmacist 7
East Midlands	General practitioner 2
South-West England	General practitioner 3
	Community pharmacist 3
Secondary and intermediate care	
Teaching hospital 1 (North-West England)	Renal consultant 2
	Renal registrar 1
	Pharmacist 3
Teaching hospital 2 (North-West England)	Pharmacist 2
Teaching hospital 3 (North-West England)	Renal consultant 1
	Pharmacist 1
Teaching hospital 4 (East Midlands)	Renal consultant 1
	Renal registrar 1
Intermediate care facility (North-West England)	Consultant physician 1
	Pharmacist 1

### Data collection

Semistructured interviews were conducted on a one-to-one basis, either face-to-face at the participant's place of work or (in the case of teaching hospital 4) by telephone. All interviews in the primary care sites were conducted by RLM, a postdoctoral primary care researcher, between May and September 2014, and all interviews in the secondary and intermediate care sites were conducted by DLP, a postdoctoral patient safety researcher, between February and September 2014. As outlined previously, the primary care interviews were originally for a separate study on AKI prevention; therefore, the topic guide is different to that used with the secondary and intermediate care participants; specific topics introduced in each set of interviews are listed in box 1. To ensure that the two sets of interviews were comparable in their content, the study team held meetings during the data collection period to discuss emerging findings and identify common issues to be raised in subsequent interviews. Every participant was invited to discuss his or her general experiences of managing medicines for patients with actual or suspected AKI.

Box 1 **Topic guide for the interviews**

Primary care

How do you manage patients to prevent acute kidney injury (AKI)?

How do you coordinate with hospital staff about medications people are taking and their conditions?

What happens when someone is discharged from hospital? What information are you given and who is this from?

How is this coordinated between the hospital and your practice/pharmacy? How do you coordinate with the local pharmacists/general practitioners?

How do you coordinate changes in medicines? What are the implications for you managing and restarting medicines?

Secondary and intermediate care

What do you have to do in order to achieve your objectives? Who or what is involved?

What is your experience of working with AKI patients?

With regard to the transfer of patients between primary and secondary care:

- What is the process for doing this?
- How do you deal with medication issues?
- How well does it work in practice?
- What problems do you encounter, and how do you overcome them?

Each interview was audio-recorded and transcribed with the participant's permission, and lasted for between 13 and 107 min depending on the participant's availability, with an average length of 37 min. Each participant gave informed consent prior to being interviewed, and all interview transcripts were anonymised prior to analysis. None of the participants were available to review their transcripts.

### Analysis

We analysed the interview transcripts thematically using the template method.^38^ V.10 of the NVivo software program was used to document the analysis. To create the initial template for the analysis, DLP read through all of the transcripts and noted the general themes that recurred across them. Consistent with the realist approach outlined previously, these themes reflected the people, artefacts and interactions that had an effect on medicines management in AKI. The template thus created was developed further by DLP, by reading through the set of transcripts again and comparing the content of the template to that of the transcripts. Where additional themes or subthemes emerged on rereading, these were added to the template; themes that became redundant with the addition of new themes, or whose meaning became different on rereading of the transcripts, were removed. The process of rereading transcripts and updating the thematic template continued until no further modifications were made to the template. The themes were then reviewed by RLM and the other authors (TB, a general practitioner, and DMA, a pharmacoepidemiologist, neither of whom had been directly involved in data collection or the data analysis) to ensure that they adequately addressed the subject matter and accounted for the content of the transcripts. The themes were also reviewed by one of the study participants (a hospital pharmacist), a member of the sampling frame who had been unavailable for interview (a renal consultant) and a healthcare professional from outside the sampling frame but with knowledge of the subject matter (an AKI specialist nurse). Finally, the themes were compared with the content of two pilot interviews that had been conducted with hospital pharmacists prior to data collection.

## Results

**Figure 1:**
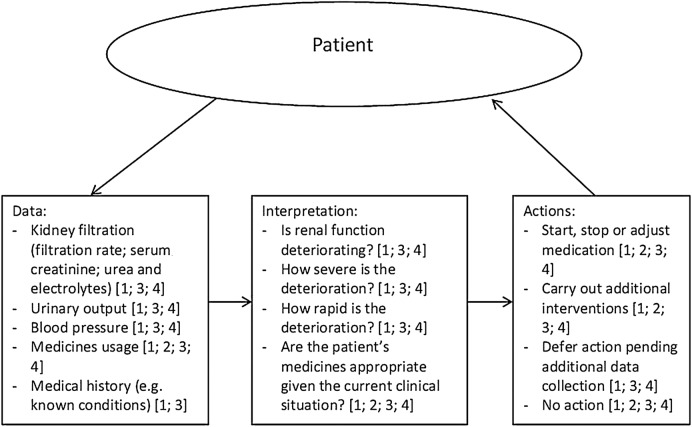
A representation of the work involved in managing patients when acute kidney injury may be present. Key: ‘1’ indicates the involvement of primary care doctors; ‘2’ indicates community pharmacy; ‘3’ indicates secondary care doctors and pharmacists; ‘4’ indicates intermediate care doctors and pharmacists.

Figure 1 provides a basic representation of the work involved in managing a clinical situation when AKI may be present. It depicts a cycle between gathering data from the patient, interpreting the data and then taking actions based on one's interpretation of the data (see table 2 for a glossary of relevant technical terms). As the figure shows, the work is distributed between the patient, community pharmacists, primary care doctors, hospital doctors and pharmacists, and (when a patient is transferred from a hospital to an intermediate care facility for further observation prior to being discharged to home) intermediate care doctors and pharmacists. In general terms, the patient journey during actual or suspected AKI may take one of the following forms:

- The patient presents to the primary care practitioner (general practice doctor or community pharmacist) with symptoms of AKI (feeling unwell with a history of renal impairment, or showing a change in glomerular filtration or serum creatinine as described in table 2). These symptoms are managed within primary care by medication changes (typically reducing or stopping angiotensin-converting enzyme inhibitors, angiotensin receptor blockers, non-steroidal anti-inflammatory drugs, diuretics and metformin, unless there is a clinical indicator for their continuation) and self-care by the patient (staying hydrated).
- The patient presents to a primary care practitioner with symptoms that cannot be managed in primary care. The patient is therefore referred to the hospital, where medication is changed and additional investigations or interventions (such as kidney biopsy or dialysis) carried out as necessary. The clinical specialty that takes primary responsibility for the patient may be nephrology; however, if the patient has comorbidities, it is quite possible that a different specialty such as cardiology will oversee the patient's treatment. Once the patient is stable and fit for discharge, he or she is then discharged to primary care, with the primary care practitioner then carrying out follow-up tests and medication changes. The patient may be treated entirely in primary care, or attend hospital outpatient clinics as required.
- The patient attends hospital with a different clinical presentation (typically dehydration or urinary tract infection), but subsequently develops or is found to have AKI. In other words, AKI is a secondary diagnosis. The patient is then treated as in (B).
- The patient is treated in the hospital as in (B) or (C), but following treatment is not well enough to be discharged directly to the community. Therefore, the patient is instead transferred to an intermediate care facility, where doctors or pharmacists may make changes to medication prior to discharge.

**Table 2:** Terminology relating to AKI management

Term	Definition
ACE inhibitor	A class of drug used to treat hypertension
GFR/eGFR	A measure of renal function. For an acutely ill patient, a GFR value of <60 mL/min/1.73 m^2^ or a rapid decrease of GFR indicates a risk of AKI*
Serum creatinine	A by-product of kidney functioning that may be detected in a blood test. An increase in creatinine level of 26 µmol/L or greater within the previous 48 hours, or an increase of 50% over the previous 7 days, confirms AKI*
Urea and electrolytes	By-products of kidney functioning that may be detected in a blood test

*Criteria obtained from NCGC^22^.

ACE, angiotensin-converting enzyme; AKI, acute kidney injury; eGFR, estimated glomerular filtration rate; GFR, glomerular filtration rate.

During the patient journey, various information sources (listed in table 3) were used to communicate current medication usage and recommended or already implemented changes to medication. The choice of source depended on the circumstances of the patient's transfer and the information that was available to the practitioners involved.

**Table 3:** Sources of information about medicines usage and changes

Pathway	Information source
Primary to secondary care	Prescription form
	Letter or note from the general practitioner
	Letter from a hospital clinic
	Printout of patient record
	Medicines administration record (from care homes)
	Verbal handover
	Medicines dispensing system (eg, dosette box)
Secondary to primary care	Letter or note from the hospital
	Discharge form

Table 4 summarises the final set of themes obtained from the template analysis. The themes in the template are described as follows.

**Table 4:** The thematic template

Theme	Subthemes
Clinical context	Assessing renal condition
	Assessing drug effects
	Trading-off effects
Organisational context	Assimilating data across organisational boundaries
	Coordinating care activity between settings
	Involvement of ‘outside’ organisations
	Limited communications
Meeting the challenges	Using alternative data sources
	Patients as mediators of collaborative work
	Adapting the system of work

### The challenges of managing medicines in AKI

#### The clinical context

With the range of possible causes and comorbidities, and the various ways in which it can manifest, AKI is an inherently complex syndrome to detect and manage. Renal function is observed primarily through a set of physiological markers, such as blood pressure, urinary output and serum creatinine levels. However, each of these markers gives only an indirect and incomplete indication of current renal functioning, and a healthcare practitioner may have to make a judgement about what can be read into a given marker given the patient's general well-being and medical history (eg, whether the patient has a chronic renal condition that reduces baseline kidney function).

I've got a lady [who] has probably had chronic kidney disease for a while, but [I don't have a baseline for her], then you've suddenly got a GFR that's in the mid-40s. [But] she's completely […] well. […] As opposed to someone who's unwell and has got a very reduced GFR, then […] you're going to do something different, really. It depends on how they're feeling [and] what clinical state you think they are. [If] […] you're not quite sure […] [then the] only way of actually knowing [is to] increase the monitoring, [to get] an idea on a clinical state. [Doctor, General practice]

Furthermore, the presence of comorbidities introduces the possibility that taking a particular action to manage AKI (eg, suspending medication that is contraindicated given the patient's comorbid condition) leads to suboptimal management of the other problems, or vice versa. Participants in our study described managing medicines in the presence of AKI as an iterative process of changing or maintaining the regimen, assessing its effect on well-being and trading-off different effects according to one's belief about what is best for a given patient.

For instance you might have a patient who has come in with an AKI and they're on furosemide, so normally you might think, […] let's stop that furosemide, […] let's reduce the dose, but that patient might also have severe heart failure and they might be short of breath, […] they might still need the diuretic, so it's not as simple […]. You might have to say let's cautiously give it and monitor things like fluid output, urea and electrolytes, just to make sure it's not doing any [more] harm. [Pharmacist, Teaching hospital 2]

As these accounts show, the technical aspects of medicines management in the presence of AKI can be challenging in themselves. Further challenges arise, however, when the task requires a practitioner in one setting to make use of information from a different setting, or to allocate actions to practitioners in a different setting. In those circumstances, the management of patients with AKI can cross professional and organisational boundaries, the implications of which are discussed in the following subsection.

#### The organisational context

For many of the participants, identifying the clinical situation or executing a plan of action required input or cooperation from elsewhere. This is often a result of the patient being transferred between primary and secondary care. For example, if a patient is transferred to secondary care in the course of managing the AKI episode, hospital staff will need to understand the patient's medical history, renal functioning and medicines usage while in primary care, in order to assist with their own decision-making. Within secondary care, information about medicines usage needs to be shared between the doctors and pharmacists who are involved in assessing or treating a patient. For example, depending on how AKI manifests, a hospitalised patient may move between the emergency department, renal specialists, cardiac specialists and a general ward. While information transfer within a hospital is unaffected by interorganisation boundaries, there may still be intraorganisation boundaries between wards and specialties that disrupt information flow. As one of our participants (a pharmacist in an acute admission unit) explained, this situation places a particular onus on staff to be vigilant about information handling.

It's easier to guarantee good flow of communication [if the patient remains] on your own ward, as you've only got your documentation. […] If it goes from another ward that slightly makes things a bit more difficult because it's […] another area, it's another pharmacist. I think it [also] depends on how long the patient's in for because […] you might only have a week to two weeks on [a] drug chart, which is then completely transcribed, so you increase the risk of transcription errors there. As time goes on more changes might be made which might then get missed [from the documentation]. […] Another factor is the doctor that's writing the discharge, how thoroughly have they gone through the notes. If it's a big pile […] they might miss things. […] But then equally if you communicate the information to them they'll have that documentation and then they can then communicate that to the GP or whichever primary healthcare provider. [Pharmacist, Teaching hospital 2]

When the patient is transferred from secondary to primary care for post-AKI care, doctors and community pharmacists (and possibly intermediate care staff, if the patient is initially discharged to intermediate care) will need to know what has happened to the patient while in secondary care and what follow-up actions are required of them, most notably with regard to continuing or changing the medication regimen and carrying out any patient monitoring. Within primary care, doctors and pharmacists (who work separately from each other) will need to ensure that they have a common understanding of the patient's medicines usage and medication needs.

In practice, the discharge information (which, as given in table 3, can come in different forms) arrives in primary care through different routes: by post; by fax; by email or hand-delivered by the patient. Sometimes, the data needed to identify and manage the situation were readily available and of suitable quality; but, on other occasions, participants encountered difficulties in assimilating or making use of data, either because it was not immediately accessible or because it contained ambiguities or inaccuracies. As the following excerpts show, either primary or secondary care practitioners could encounter such difficulties.

Sometimes [I cannot tell from] the discharge whether the hospital [has administered] all the tablets that [the patient is supposed to be] on. It's very clear on the discharge which ones they've stopped, but you've got all these other tablets and you think, have they stopped them or have they just not been restarted. […] So then you're having to chase up the hospitals to find out whether that has actually been done. […] [But] you speak to the secretary who then says, I'll contact the consultant, […] there's a couple of days' delay there and the patient's running out of medication, so you've [then] got to make the decision [yourself]. [Doctor, General practice]

[To get the patient's medical history on admission,] we […] have to contact the GPs directly […] and get faxes [sent]. […] It's time-consuming. [And] trying to define whether the patient [normally has] chronic renal impairment […] can take extra time because the receptionist will have expanded drug history facts that they will send back; but if you're saying, could you get me [the previous urea and creatinine], […] that will take longer. […] It usually means a second call as well, because [the technician has] already phoned and requested [for basic information]. [Pharmacist, Teaching hospital 1]

These participants' accounts allude to the effect of organisational boundaries between primary and secondary care. These serve to close off the required data within different organisations, each with its own way of storing, accessing and using the data. The participants describe having to negotiate access either to another organisation's data or to people there with relevant knowledge about the case at hand. In either case, the participant did not get direct access, but instead have to work with an intermediary (eg, an administrator). A second issue identified in these excerpts was that the data obtained was presented in a manner that made sense to the originating organisation, but not necessarily to the recipient, who did not have the contextual knowledge needed to interpret the data. In addition, there was temporal and physical separation between the organisations, such that communication between practitioners lagged behind the progress of the patient. As a result of these issues, the task became less efficient than it might be, with primary and secondary care practitioners spending additional time and effort trying to resolve ambiguities or gaps in the data, and struggling to keep up with the timelines for follow-up actions.

[We get] the patient [going] into hospital and then [coming] out with no information. Typical discharge letter [says], Mrs Brown's come out of hospital, please check her U&Es in three days. Well first of all, the letter's undated so you don't know when three days is. Secondly, you've only just received the letter, even though the patient's been out for five days. And thirdly, you don't know what the U&Es were before you started. [Doctor, General practice]

When we ask the GPs to take some action or when we inform them of what we've done, it does appear sometimes that that doesn't happen. [They] often say that they don't receive [the information] in time. Often we say repeat U&Es in one or two weeks and take some action and the GP will say, I didn't get the discharge until three weeks later so how was I to know? [Pharmacist, Teaching hospital 2]

In some cases, particular organisations or departments (eg, community pharmacies and specialist clinics) had an important role in patient care, but were not routinely involved in communications about patient admission and discharge. This can cause additional complications with data gathering or the coordination of care activities, further increasing the workload for all involved and the risk of interventions being missed or delayed.

Participant: We don't always hear about [a patient having AKI]. We don't see discharge summaries and things like that, so you don't necessarily know.

Interviewer: How do you find about changes to [a patient's medication]?

Participant: Next time the prescription turns up usually. […] For patients on blister packs it tends to be a bit better in that the local hospital [phones] us up and [tells] us [about any changes]. [Pharmacist, Community pharmacy]

We had an instance recently where a patient was discharged from us and a copy of the discharge summary sent to the community pharmacist and sent to the GP. But when I spoke to the pharmacy about this patient, four [prescription] items were missing. The community pharmacist had apparently requested items from the GP and rather than looking at the discharge summary, the GP had just actioned the items the pharmacy here had requested. I went back to both of them and [asked them why]. [The pharmacist] had thought that the GP provided the stuff that they wanted the patient to be on. And the GP thought that the chemist had requested what the patient should be on. So the patient ended up without her rheumatoid arthritis medicine [or] her beta-blocker. [Pharmacist, Intermediate care]

The experience of the intermediate care pharmacist is noteworthy, as it illustrates an apparently unsuccessful attempt to improve coordination between different organisations. While providing discharge details to the primary care doctor and the community pharmacist might be a sensible intervention in principle, it depends on the ‘end users’ having the capacity to use this information effectively. In this case, it appears that a lack of communication between the two parties forestalled any expected benefits. Other participants similarly described being relied on to make an imperfect system work effectively, with varying degrees of success.

Sometimes the patient has come in with a list of medicine because […] they saw their GP [who] said […] they're really unwell, let's send them to hospital, sometimes they're really good and […] print out a brief summary which has got all the list of medicines on, so we like it when they do that. But some of the time they don't. So I don't know whether they're aware that we don't have that information already or whether they think that someone else will sort it. [Pharmacist, Teaching hospital 1]

The information we're given is very variable, in [terms of] its usefulness. […] You're getting, [with no context], “please do the [this patient's] U&Es”. Yes, I'll do them, [but] what do you want me to do with them? I've never seen one with a plan, they just say do the U&Es in a week, do a full blood count in a week […]. [That] implies you'll know what to do with the result, well maybe I don't. [Pharmacist, Community pharmacy]

In the first account, data that would help other participants fulfil their role in the process are not shared with those participants. In the second, communication occurs but it does not achieve the required effect, because the recipient does not have enough information about the task requirement. The effect of both interactions is to limit the effectiveness of collaborative working in the respective situations.

That medicines management in AKI is challenged by its technical complexity and its distribution across different settings raises the question of how it is achieved in practice. The following subsection describes ways in which participants deal with the challenges encountered during an AKI episode.

### Meeting the challenges of care management

As outlined previously, a crucial part of managing episodes of care complicated by AKI is developing an understanding of the clinical situation; yet, this understanding is often grounded in dispersed, incomplete or unavailable data. Some participants discussed the use of alternative data sources to help them make inferences in the face of information gaps.

[If details are missing from the discharge form], you [then] hope that the pharmacist [or] the district nurse has got any information and [can pull it] together. [Doctor, General practice]

[Patients] at a care home […] [have a] medicine administration record. […] They're a good source of information because […] the GP list is a list of medicines the GP wants the patient to be on but if they're in a care home and you've got that medicine administration record, then you can see where the nurses have signed to say the patient actually had the medicine. [Pharmacist, Teaching hospital 1]

The different information sources referred to by these participants (other healthcare professionals with knowledge of the patient; previous hospital records) may well help to address any ambiguities. However, as shown in the previous subsection, it is quite possible that discrepancies exist between these sources; for example, a patient's actual medicine-taking being different from that implied by a GP's or hospital's record. Therefore, practitioners make a judgement about how authoritative each source is, and if possible cross-check them, as the hospital pharmacist explains. Another hospital pharmacist described relying on the patient to help address information gaps.

[Out of hours access to GPs' data] can be very tricky. You are [then] reliant on the patient being au fait with their meds. A lot of patients carry their [repeat prescriptions]. [For] regular attenders at the hospital, we can access [hospital prescription records], so if one was within [the last three months], we can use [it] as the starting point for drug history. [For those patients] we've got old […] results [of renal function tests], as well. But if you've got somebody new, it can be tricky. So the pharmacist has got to prompt a lot, and say, do you get any meds other than from the GP? Anything delivered to your house? Do you get…you know, because sometimes things can be missed, and the patient might not think about it. [Pharmacist, Teaching hospital 1]

As a constant part of the process, and the person with the most extensive experience of his or her care interventions, the patient was also seen by other participants as an important resource. Some participants saw patients not only as a passive information resource, but also as a mediator of the collaborative work.

Sometimes the patient brings the copy [of their discharge] in, it tends to be because we highly manage those people they know to tell us. We could be caught in the middle between the patient and the GP when they come out, so we might be the first people they see. [Pharmacist, Community pharmacy]

Participant: Maybe the ACE inhibitors for instance, you stop them [but] it doesn't get across to the GP and then you might see [the patient] in clinic. You send letters to the GP asking them to change medications [but] it never happens.

Interviewer: So how does that problem get dealt with in clinic?

Participant: You have to write again or call him up. Or give a handwritten note to the patient. Sometimes that works. [Doctor (Registrar), Teaching hospital 1]

While these participants suggested coopting the patient as a messenger between practitioners, this appeared to be an ad hoc measure, adopted when other methods of coordinating activity had failed. It relies on patients being willing and able to play a role in coordinating their care, which (as some participants noted) is the case for some patients but not others. An alternative approach described by some participants was for practitioners to adapt their own methods of working to achieve better coordination. For example, one pharmacist described how he ensured that information is adequately communicated to primary care professionals, hence contributing to a collective understanding of the task state.

[Our discharge form has a] section [where you] sign your name and there's a small box beside the signature for clinical notes. I find that it's not easy to read and I wonder whether people do read it, because a few patients that I've seen where notes have been put in this box, advising the GPs to restart the medication and then they come in three months later and this medication isn't started. [However, the doctors] have a big section in the main part of [the form] which I tend to take [instead], because the doctors tend not to use it. I reference who I am and write medication to use, and then list all the tablets that have [been] stopped and started. […] The renal consultants and registrars have said that is quite useful for them […] but it's never been rolled out or anything like that. […] Maybe it's just renal [that's] a high risk area, that's why they need a bit more [detail about the medicines] [Pharmacist, Teaching hospital 1]

A primary care doctor described bringing in his own domain expertise in order to ensure that (assumed) collective task requirements are met.

I just keep marking [the list that] I've got, ticking the boxes […], so [the] medications are already there, and when [there are any changes to the medication], I just [make them here]. And if I think they have changed something, like they have added an ACE inhibitor, or done anything which might have caused any damage to the kidneys, I actually repeat the [U&E test]. Sometimes they ask us [to repeat the test] as well. [Doctor, General practice]

Another notable feature of these accounts is that they describe adaptations that the participants have undertaken locally, on their own initiative. For example, the pharmacist who used the discharge form in a non-standard manner added that he is the only pharmacist in his hospital to make this adaptation, and that it was likely to be something required only for renal patients due to the complexity of their medication.

From the accounts in this section, participants were seen to use a range of approaches to overcome the technical and organisational challenges of managing AKI. Some were employed on an opportunistic basis as circumstances allow it, while others were employed more consistently.

## Discussion

The challenge of optimising medicines in the presence of AKI is twofold. Intrapatient and interpatient variation in kidney health, and its interaction with other aspects of well-being, makes detecting AKI and taking action to deal with it technically complex. It is made more difficult by the physical and temporal distribution of the work across different practitioners, staff and patients, based on different organisations; this causes problems for the sharing of information and coordination of activities that are required to ensure effective collaborative working. Participants described ways in which they achieve collaboration, which involve employing alternative means of communication or sources of information, or adapting their own working methods.

The current study highlights how the interactions between primary and secondary care can be seen as an instance of distributed cognitive work. From this viewpoint, a few observations might be made about medicines management in the context of AKI. First, the work relies on a number of cognitive functions that take place within and across organisational settings. These include decision-making, which depends on the ability to identify and interpret data and evaluate the effect of any actions, and coordination, which is achieved by a mixture of implicit and explicit methods. That these functions are involved suggests that they (in addition to purely technical knowledge) should be a focus of any improvement interventions. For example, interprofessional training events and decision aids could be designed to foster the knowledge and skills required to coordinate medicines management activities. A second, more specific, point concerns the role of communication. In the context of collaborative work, there is more to communication than possessing and transmitting knowledge, it is a means by which collaborators interact to develop shared understanding.^39^ ^40^ This view is reflected in our interpretation of some situations recounted by participants here, in which communications took place that fulfilled the obvious purpose of passing on data, but nevertheless left the recipient somewhat uninformed. Presumably, communication strategies that address themselves to the development of collective knowledge about the situation at hand will help to avoid this problem and, hence, could be encouraged by the design of communication aids.^15^ ^41^ The final observation is the situated nature of some of the work adaptations described by participants. In other words, the adaptations were for specific organisations or practitioners rather than others, and possibly also for care complicated by AKI as opposed to other scenarios. Wears^42^ notes that the potential need to adapt work activities in light of situational circumstances should be taken into account when deciding how much to standardise practitioners' activities. Indeed, there may be a learning benefit from considering ways in which practitioners have adapted work practices. On the other hand, though, it is important to ensure that any adaptations made by one person or group do not cause difficulties for others elsewhere who are also involved in the work. Furthermore, it may be the case that improvisation by frontline staff is symptomatic of shortcomings in the design or implementation of the work system, and that addressing these shortcomings would be preferable to relying on the initiative of staff members to maintain the system functioning.^20^ One design intervention is the provision of shared access between primary and secondary care professionals to a patient record. In principle, this would help them to establish a common understanding of medicines usage, which will provide a basis for coordinating work activity between organisations. However, given the foregoing observations about communication, some thought would need to be given to how information is presented to different users according to their needs. Also, previous studies suggest that a combination of interventions, rather than a single intervention, would be needed in order to make an appreciable improvement to medicines management across care boundaries.^43–46^ For example, one outstanding issue identified in this and previous studies would be the alignment of task schedules and staff availability between the different organisations or specialties in order to streamline cross-boundary working.

Our findings support the notion that examining the work involved in a distributed clinical activity, paying attention to how it is shaped by the organisational contexts across which it occurs, can provide insights into what is needed to ensure those activities are carried out successfully. In theoretical terms, they demonstrate both the role of cognitive factors in technical and collaborative work, and a perspective on quality and safety that recognises the contribution of practitioners and service users to the effective functioning of a complex, high-risk system.^47^ ^48^ In practical terms, our findings inform interventions to improve medicines management. Specifically, they highlight the need to incorporate support for decision-making, communication and coordination as part of such interventions, whether through training, procedures, staffing or tools.^48^ ^49^ Our study focused on episodes of care that are complicated by AKI; however, the activities that are involved, and the issues that arise with regard to these activities, are ones that are likely to be present in other clinical scenarios. For example, our findings may be relevant to any clinical activities that involve the discharge of patients from secondary to primary care, or the coordination of care between general practice and acute or non-acute providers, both of these having been identified as warranting efforts to improve their effectiveness.^50^ ^51^

By examining practitioners' regular experiences of managing AKI, this study complements case note and incident reviews that focus on the process and outcome of specific episodes. The qualitative approach allowed us to explore, in depth, the issues that participants encounter, and how they deal with these issues. This, in turn, provides an insight into the behaviours and circumstances that support effective management of the clinical situation as well as those that may be implicated in adverse events. However, while our study benefits from a sample that represents practice in different areas of England, it is possible that differences between or within countries in the organisation of medicines management or kidney care limit the extent to which our findings represent care settings in other locations. In settings where medicines management activities occur entirely or predominantly within one organisation, or there is closer integration between the different organisations involved, some of the issues identified here will be less problematic. Despite the breadth of our sample, we cannot rule out the possibility that participants self-selecting into the study, and the channels by which we identified potential participants in the first place, have introduced bias into the views of the sample regarding the study topic. Finally, resource limitations meant that we were unable to collect any observational data, which would have allowed methodological triangulation and an opportunity for the researchers to corroborate the participants' accounts.

Our study has found that, in the case of a clinical problem that crosses care boundaries, there are a number of technical and organisational challenges to medicines management, and that meeting these challenges involves cognitive work such as decision-making, planning and team coordination. We would suggest that future work follows two lines of inquiry. One is to further investigate how knowledge is created and used in medicines management or the management of AKI. The other is to develop and evaluate systems of work that support the activities of those involved in such tasks, as described earlier in this section.
